# Effects of a six-month intradialytic physical ACTIvity program and adequate NUTritional support on protein-energy wasting, physical functioning and quality of life in chronic hemodialysis patients: ACTINUT study protocol for a randomised controlled trial

**DOI:** 10.1186/1471-2369-14-259

**Published:** 2013-11-26

**Authors:** Justine Magnard, Thibault Deschamps, Christophe Cornu, Anne Paris, Dan Hristea

**Affiliations:** 1 University of Nantes, Laboratory “Motricité, Interactions, Performance” (UPRES EA 4334), F-44000 Nantes, France; 2 ECHO Nantes, Centre de dialyse Laënnec, 23, rue des Piliers de la Chauvinière, 448000 Saint Herblain, France

**Keywords:** Hemodialysis, Protein-energy wasting, Nutritional supplementation, Intradialytic physical activity, Quality of life

## Abstract

**Background:**

Protein-energy wasting (PEW) is common in hemodialysis patients and is a powerful predictor of morbidity and mortality. Although much progress has been made in recent years in identifying the causes and pathogenesis of PEW in hemodialysis patients, actual management by nutritional interventions is not always able to correct PEW*.* Some investigators suggest that physical exercise may increase the anabolic effects of nutritional interventions, and therefore may have a potential to reverse PEW. The aim of this study is to investigate the effect of intra-dialytic progressive exercise training and adequate nutritional supplementation on markers of PEW, functional capacities and quality of life of adult hemodialysis patients.

**Methods and design:**

Fifty end-stage renal disease patients undergoing hemodialysis, who meet the diagnostic criteria for PEW, will be randomly allocated into an *exercise* or *control* group for 6 months. The exercise consists of a progressive submaximal individualized cycling exertion using an adapted cycle ergometer, during the three weekly dialysis sessions. Biological markers of nutrition (albumin, prealbumin) will be followed monthly and all patients will be assessed for body composition, walk function, muscle strength, postural stability and quality of life at baseline and during the eighth week (t_+2_), the sixteenth week (t_+4_) and the twenty-fourth week (t_+6_) of the 6-month adapted rehabilitation program.

**Discussion:**

The successful completion of this current trial may give precious clues in understanding PEW and encourage nephrologists to extend prescription of exercise programs as well as therapeutic and as preventive interventions in this high-risk population.

**Trial registration:**

The protocol for this study was registered with the France Clinical Trials Registry NCT01813851.

## Background

Nowadays, more than two million people are treated worldwide by dialysis for end-stage renal failure [1]. Despite significant progress in dialysis techniques and in the treatment of associated comorbidities, hemodialysis (HD) patients experience an impairment of their quality of life and have a much higher risk of mortality compared to an age-matched population. A sedentary lifestyle and an altered nutritional status have been identified as major risk factors for adverse outcomes in dialysis patients [2,3].

Dialysis patients have decreased physical functioning (assessed by peak oxygen consumption ($\dot{V}O2\max$), physical performance tests, and self-reported functioning), diminished muscle mass and altered muscle quality, and all of these features are associated with an increased mortality risk [4-6]. These disturbances are directly related to renal failure and comorbidities, and also to adverse effects from medical treatments and from the dialysis itself. Dialysis induces notable metabolic changes: hypovolemia due to ultrafiltration, and rapid changes in electrolyte concentrations and systemic inflammation, which can all adversely affect physical function [7]. In addition, dialysis imposes immobilization over 12–18 hours a week, thereby directly contributing to sedentary behavior that can further worsen the medical condition of HD patients. This vicious circle can finally lead to the development of disability, loss of independence, and death [8]. For all these reasons dialysis patients have low levels of daily physical activity [9]. Therefore, it seems rational to promote programs for exercise training in this population.

In this vein, a large amount of literature published in the past 30 years has documented a myriad of potential benefits from exercise. Several include: increased maximal oxygen uptake capacity ($\dot{V}O2\max$), improved blood-pressure control [10], decreased arterial stiffness [11], decreased systemic inflammation [12], improved solute removal by dialysis [13,14], increased muscle mass, quality and force [15], and favorable psychological adaptations (e.g., higher perceived quality of life) [16]. Thus there is a large body of evidence that the impact of end-stage renal disease (ESRD) can be – for at least a significant part - counteracted by exercise training [7,17-20].

Along with this altered overall physical functioning, malnutrition is highly prevalent in the ESRD population, and is well-documented to be a strong predictor of mortality. Significant evidence comes from the data of large observational trials (USRDS, DOPPS [1-21]) showing that malnourished dialysis patients have an increased risk of mortality [22,23]. In this context, the International Society of Renal Nutrition and Metabolism (ISRNM) has recommended the term “protein–energy wasting” (PEW) to describe the loss of body protein mass and fuel reserves and have defined the diagnostic criteria for this state (see Table 1).

**Table 1:** ISRNM criteria for the clinical diagnosis of PEW in maintenance dialysis patients

Criteria*	
*Serum chemistry:*	
	Serum albumin < 3.8 g per 100 ml
	Serum prealbumin < 30 mg per 100 ml
	Serum cholesterol < 100 mg per 100 ml
*Body mass:*	
	BMI < 23
	Unintentional weight loss over time: 5% over 3 months or 10% over 6 months
	Total body fat percentage < 10%
*Muscle mass:*	
	Muscle wasting: reduced muscle mass 5% over 3 months or 10% over 6 months
	Reduced mid-arm muscle circumference area (reduction > 10% in relation to 50^th^ percentile of reference population)
	Creatinine appearance
*Dietary intake:*	
	Unintentional low dietary protein intake <0.80 g.kg^-1^.day-1 for at least 2 months
	Unintentional low dietary energy intake <25 kcal.kg^-1^.day-1 for at least 2 months

*At least three out of the four listed categories (and at least one test in each of the selected category) must be satisfied for the diagnosis of kidney disease related PEW. Optimally, each criterion should be documented on at least three occasions, preferably 2-4 weeks apart.

ISRNM, international society of renal nutrition and metabolism; PEW, protein-energy wasting; BMI, body mass index.

All these criteria are individually associated with an increased risk of adverse outcome so it can be assumed that the concomitant presence of at least three criteria is an even more powerful predictor of morbidity and mortality. However, to the best of our knowledge, until now no report exists about the prevalence and clinical outcomes of PEW (as defined by the ISRNM). It can be noted, however, that a large observational French study reported that 36% of HD patients had a prealbumine < 300 mg/dl, 20% had albumin < 35 g/l and 62% a diminished lean body mass (LBM) < 90% [24].

The mechanisms causing PEW are complex and multifactorial: low nutrient intake (sometimes due to dietary restriction), loss of nutrients into dialysate, abnormalities that stimulate protein degradation and/or decrease protein synthesis. On this last point, we can include the production of inflammatory cytokines, oxidative and carbonyl stress, endocrine disorders (hyperparathyroidism, hypogonadism, diabetes, decreased insuline/insuline like growth factor (IGF) signaling), acidosis, electrolyte imbalance, and anemia [25]. Further, low daily physical activity causes the loss of muscle mass and force and thereby directly contributes to worsen PEW. Traditional management of PEW according to international guidelines (coming from the EBPG working group on nutrition [26]) consists of dietary counseling, oral nutritional supplements, enteral feeding, and in severe cases, the use of intradialytic or total parenteral nutrition. Several studies, however, have shown that these interventions are not always able to correct PEW [27].

Simultaneously, considerable literature has pointed out the anabolic effects of physical activity. For example, both strength and endurance exercise induce transcriptional changes in genes (IGF, myostatin) favoring anabolic muscle [28]. Morevover, histologic and computed tomography studies have clearly demonstrated a decrease in muscle atrophy and an improvement of the overall muscular structure and quality (increased proportion of type II, oxydative fibers) [29]. There is also some evidence that intradialytic exercise, combined with oral or parenteral nutrition, enhances amino acid uptake and protein accretion in the muscles of HD patients [30,31]. Strong arguments exist for programming the adapted exercise training during the dialysis session rather than on non dialysis days, as there is better adherence by patients who feel safer doing exercise under medical monitoring, as a the counteraction to the inactivity due to the dialysis session, and due to a greater removal of uremic toxins (as shown for urea (KT/V) and phosphorous) by improved tissue mobilization. In addition, according to the large amount of published clinical trials, the risks of prescribing exercise even in this frail population are limited and the benefits largely prevail. Musculoskeletal injuries and cardio-vascular events are the most common risks of physical exercise. Both types of adverse events occur more frequently with high-intensity exercise than with submaximal exercise [32]. In this present trial, patients will be advised to exercise at a moderate level of perceived exertion and will be under continuous supervision by medical personnel. It should also be mentioned that European guidelines on nutrition in HD patients explicitly recommends regular physical exercise [26].

In sum, there are strong arguments in the literature to prescribe exercise in HD combined with nutritional interventions in patients suffering from PEW in an attempt to enhance the anabolic effects of nutrition and hereby reverse this high-risk state.

### Specific aims

The aim of this randomized controlled trial is to analyze the impact of a progressive intra-dialytic exercise program combined with nutritional support following current guidelines on nutritional parameters defined for PEW and its potential to reverse PEW. In addition, this trial will study the effect of intradialytic exercise on functional performance (walking, postural control, and muscular strength), body composition and health-related quality of life in HD patients.

## Methods and design

### Study design

This study is a multicenter, open-label randomized controlled trial. The study will be conducted at the outpatient HD Laennec Dialysis unit and Confluent Dialysis unit of the ECHO Dialysis Association. HD patients will be randomly distributed into a *control* or an *exercise* group. The randomization sequence of the participants will be generated by a computer program. A bio-statistician not involved in recruitment and assessment will perform the randomization.

The present study was approved by the Ethical Committee of Nantes Ouest IV (reference: ID RCB n°2012-A01662-41), and was conducted in accordance with the Declaration of Helsinki (last modified in 2004). All participants will receive written and verbal information about the aims and procedures of the study and will sign a consent form to participate in the study.

### Participants’ recruitment

All of the 210 patients treated by hemodialysis or online-hemodiafiltration in the participating centers will be screened for the presence of criteria for PEW. The principal investigators will review the existing patients’ database for serum albumin, serum prealbumin, C-reactive protein (CRP) levels*,* body mass index (BMI) and the presence of weight loss according to the PEW criteria. Potential subjects will have additional tests (measure of lean body mass index by bio-impedance and a 3-day dietary record by a qualified dietician in order to evaluate their protein and energy intake). Patients fulfilling inclusion/exclusion criteria and having signed an informed consent for the study participation will undergo randomization generated by a computer program. We expect to recruit approximately fifty HD patients for this study.

### Inclusion criteria

•Adult patients, male or female (Age > 18 years).

•Minimum hemodialysis vintage of 3 months.

•Stable on HD, in gender.

•No recent hospitalization.

•No acute or chronic medical conditions that would make exercise training potentially hazardous or primary outcomes impossible to assess.

•Patients who meet the following criteria for PEW (according to [25]), meeting at least three out of the four listed categories and at least one test in each of selected category:

– *Serum chemistry criteria*: Serum albumin level < 38 g/L (Bromcresol Green), or serum prealbumin < 300 mg/L,

– *Body mass criteria*: BMI < 23 kg/m^2^, or unintentional weigh loss > 5% over 3 months or > 10% over 6 months,

– *Muscle mass criteria*: lean body mass (LBM) estimated by bioimpedance spectroscopy (Body Composition Monitor (BCM) Fresenius, Bad Homburg, Germany) lower than the 10^th^ percentile of an aged-matched normal population. This method is validated in dialysis patients [33,34].

–Dietary intake criteria: Unintentional low dietary protein intake < 1 g/kg of ideal weight/day for at least 2 months, unintentional low dietary energy intake < 30 kcal/kg of ideal weight/day for at least 2 months.

•Informed consent of the patient.

### Exclusion criteria

•Contraindication or inability to perform the physical exercise.

•Inadequate dialysis Kt/V < 1.2.

•Presence of a cardiac pacemaker (incompatible with the BCM measures).

•Systemic inflammation CRP > 20 mg/l.

•Pregnant woman.

•Patient under guardianship.

•Participation in another clinical interventional trial.

•Unstable on dialysis.

### Intervention

All the patients will continue their usual dialysis procedure (HD or HDF). No modifications of dialysis modality or prescriptions are allowed except for adaptation of dry weight using clinical criteria.

Both patients in the control and exercise groups will receive dietary counseling by a trained dietician. The prescription of oral nutritional complements or intradialytic parenteral nutrition will be adapted to patient needs according to the dietary record in order to attain goals set by the EBP guidelines for gender energy intake 30–40 kcal/kg of ideal weight/day, and protein intake > 1.1 g/kg of ideal weight/day.

### Exercise group

A 6-month adapted rehabilitation program will be conducted by means of progressive submaximal individualized cycling exercise, consisting of three sessions per week. The exercise will be prescribed during the first two hours of dialysis session using an adapted cycle ergometer (« Reck moto-med letto ») that allows cycling in a supine position at different resistance levels. The cycle ergometer displays the number of revolutions per minute, the power developed and the distance virtually traveled. For the patient, these types of visual feedback are motivational factors that are recognized in the literature [35]. Further, it should be noted that this cycle ergometer also allows for passive motorized pedaling (i.e., the patient can pedal without effort).

The aim of this 6-month individualized program is to reach a 30 min duration of continuous cycling at a moderate exercise intensity, which corresponds to the level “3 – moderate” on the Borg Rating Perceived Exertion (RPE) scale (graduated from 0 to 10, where 0 = no effort at all, and 10 = most extreme effort imaginable). According to recommendations issued from a large scale meta-analysis [36], the exercise will be initially performed at this intensity exercise perceived as moderate by the patient on the RPE scale (i.e., level 3). We deliberately chose this method based on the patient’s rating perceptions rather than a method based on the heart rate assessment because many dialysis patients have automatic impairment or drug treatments that can interfere with the heart rate control. This method is readily accessible, reproducible and easy to use. Several exercise training intensity levels at 50% to 85% of the maximal oxygen consumption correspond consistently to ratings of 3–7 levels on the Borg RPE scale [37].

To estimate the targeted moderate exercise intensity, an exercise protocol with an increasing intensity on the cycle ergometer will be performed at the inclusion, before the rehabilitation program. During this first evaluation, after a warm-up of 5 minutes without resistance, the patient will pedal with a self-selected chosen cadence against an increasing resistance imposed by the cycle ergometer, until the patient reaches a level perceived as moderate, namely level 3 on the Borg RPE scale. A similar monthly assessment will be realized to update the intensity of cycling exercise. During these sessions, the patient will be regularly asked about level of shortness of breath, any feelings of fatigue and pain, with a visual analogue scale (0–10 cm), in order to reduce the exercise intensity if necessary.

During a dialysis session, the cycling exercise will be performed in the first half of the session. After a 5 min warm-up without resistance, the patient will gradually reach the intensity corresponding to level 3 on the Borg RPE scale. Then the patient will be instructed to maintain this cycling exercise intensity for at least 10 minutes during the first month, 15 minutes during the second one, 20 minutes for the two next months in order to achieve the targeted exercise duration of 30 minutes in the two last months. The first target is to observe an evolution of their functional capacity/performance over time. Finally, a period of active recovery (pedaling without resistance) followed by a period of passive pedaling recovery will end the exercise session. Blood pressure and heart rate will be measured at the beginning and the end of the exercise session. Heart rate will be maintained below 70% of maximum heart rate during the cycling exercise. At the end of the dialysis session, the HD patient will be asked to estimate pain level, boredom level and mood as perceived during the session, with a visual analogue scale graduated from 0 to 10. Nephrologists, nurses, and a specialist in adapted physical activities will monitor the patient.

### Control group

Similarly, at the end of each dialysis session, the “control” patient will be asked to estimate his pain level, boredom level and mood perceived during the session, with a visual analogue scale graduated from 0 to 10 [38].

### Outcome measures

All the measures listed below will be assessed at the baseline t_*0*_, and during the eighth week (t_+2_), the sixteenth week (t_+4_) and the twenty-fourth week (t_+6_) of the 6-month adapted rehabilitation program.

### Primary outcome measures

The primary outcome of this study is the number of patients who are no longer in a state of protein energy wasting after 6 months of exercise training compared to the control group. We define correction of PEW as the normalization of albumin and prealbumine, and of the lean tissue index, considering these items to be the clinically most relevant for the diagnostic criteria of PEW.

#### Biological sample

Patients in both groups will have routine serum chemistry measures according to follow-up guidelines for dialysis patients in terms of serum electrolytes, -bicarbonate, s-urea and creatinine, urea reduction rate, KT/V monthly, PTH and 25 OH vitamine D, which will be measured at the beginning and at the end of the study. Patients will receive oral native vitamin D supplementation in order to maintain serum 25 OH levels in the normal laboratory range.

#### Dietetics survey

Protein and energy intake will be assessed by a qualified dietitian by means of a 3 day food record at the beginning and at the end of the study.

### Secondary outcome measures

Secondary outcome measures include: (i) changes in biological markers of nutrition (albumin and prealbumine), body composition (LTI, FTI), muscle strength, walk function, postural stability, quality of life, days of hospitalization, and survival.

#### Biological markers of nutrition (albumin and prealbumine)

Serum albumine and -prealbumine will be measured monthly. BMI will be calculated at month t_+2_, t_+4_ and t_+6_. Serum albumine is measured by the bromcresol green method (normal range (38–52 g/l), serum prealbumine is measured by the immune-turbidimetric method (normal range 300–400 mg/l).

#### Body composition

Body-composition (overhydration, lean tissue index (LTI, kg/m^2^) and fat tissue index (FTI, kg/m^2^) will be measured by bioimpedance spectroscopy using a Body Composition Monitor (BCM, Fresenius Medical Care, Bad Homburg, Germany) at month 2, 4 and 6 just before the midweek dialysis session.

#### Walk function

The walk function assessment will be performed using a 6 min-walk-test (6MWT). This test has been considered to be an appropriate test to assess a patient’s functional and physiological response and cardiovascular fitness [39]. Patients will walk along a measured circuit (15 m), and are instructed to cover as much distance as possible in the six minutes. Blood pressure, heart rate and rating perceived exertion (RPE) will be assessed before and after the 6MWT.

#### Muscle strength

Maximal quadriceps strength during a knee extension will be tested using a manual dynamometer (Metil Industry©, Belgium). Patients will perform three maximal leg extensions in sitting position before a dialysis session. At least five minutes of recovery will be provided between 2 consecutive measurements.

#### Quality of life

The self-reported quality of life will be evaluated using the Medical Outcome Study Short Form 36 (SF-36) [40-42], which has been documented to be reliable and valid in dialysis patients [10,43]. This SF-36 is a generic multidimensional instrument consisting of eight multi-item scales representing: (1) physical functioning (extent to which health limits physical activities, such as self-care, walking, and climbing stairs), (2) social functioning (extent to which physical health or emotional problems interfere with normal social activities), (3) role-functioning physical (extent to which physical health interferes with work or other dally activities), (4) role-functioning emotional (extent to which emotional problems interfere with work or other daily activities), (5) mental health (general mental health, including depression, anxiety, behavioral and emotional control, and general positive effect), (6) vitality (feeling energetic and full of pep rather than tired and worn out), (7) bodily pain (intensity of pain and effect of pain on normal work, both inside and outside the home), and (8) general health perceptions (personal evaluations of current health, health outlook, and resistance to illness).

#### Postural stability

Balance during a quiet stance will be tested after a rest period before the dialysis session. The postural test will consist of two conditions of quiet stance: stance on a firm surface with eyes open (EO) and stance on a firm surface with eyes closed (EC). Participants will stand quietly while barefoot, with the head in a straight-ahead position, their arms along the body. During conditions with eyes open, they will be instructed to look at a black spot (with a diameter 2 cm diameter) placed on a white wall in the front of them at a 2 m distance. For each condition, two trials will be performed. The duration of each trial will be 60 s followed by a rest period of 1 minute. During thee condition with EO, the subject’s eyes will focus on a stationary eye-level visual target (a black spot with a 2 cm diameter) situated at a 2 m distance. For all data collection of postural sway, we will use a Kistler force plateform (model 9286BA) with subject weight normalization. Data will be sampled at 100 Hz and recorded online. The body sway will be quantified by displacement of the center of foot pressure (COP) in the anterior-posterior (AP) and in the medial-lateral direction (ML). For measuring all of the COP parameters, raw data will be filtered using a fourth-order Butterworth, zero-phase low-pass at 10 Hz. The COP parameters will then be the mean and standard deviation (SD) of COP position in AP and ML directions, SD of velocity in AP and ML directions, mean velocity and area (90% confidence ellipse) (see [44] for details of formulas).

### Statistical analysis

The sample size was calculated considering the number of patients who are no longer in a state of protein energy wasting after 6 months of exercise training compared to the control group. For a significance level of 0.05 and 80% power, it was estimated that 22 participants would be required in each group.

Results will be expressed as minimum to maximum or percentage of change, as applicable, and mean ± standard deviation. To test the effects of Group (Exercise *versus* Control) on all outcome measures, univariate two-way analyses of variance (ANOVAs), with the Group (×2) as between-subjects factor and Time (t_0_, t_+2_, t_+4_ and t_+6_) as the within subject factor, will be carried out for each aforesaid dependent variable. For each analysis, the level of significance was p < 0.05. Newman-Keuls comparisons will be used for post-hoc tests following significant effects. If the sphericity assumption in ANOVA is violated (Mauchly’s test), corrected tests of significance will be used [45,46]. In that case, the paired t-tests with corrected alpha level will be used as post-hoc comparisons. All the statistical analyses will be conducted using a SPSS software package.

## Discussion

To our knowledge, this is the first trial investigating the impact of intradialytic exercise combined with an individualized nutritional support on protein energy wasting. It is also the first trial to investigate the influence of exercise on postural balance, which is of special interest for fundamental and clinical purposes (i.e., the important issue of physical condition in dialysis patients). An improved postural balance signifies a decreased incidence of falls and fractures in this high-risk population [47]. The main forces of this trial will be the randomized design and the individualized management of “real life” patients suffering from PEW. It can be argued that the nutritional support will not be the same in all patients as some will have only nutritional counseling and others ONC and a few IDPN. However, according to the literature the different nutrition methods have equivalent efficacy and the prescription of the type of nutritional support will follow actual guidelines. If this current clinical trial can highlight the expected effects of exercise to reverse PEW, at least for a part, it would be a strong argument for implementing an exercise program in this high-risk population and even in the whole dialysis population in order to prevent the occurrence of PEW.

## Competing interests

Authors declare that there is no competing interest.

## Authors’ contributions

DH has conceived and designed the research protocol, DH and AP contributed in patients’ inclusion–exclusion criteria and submission of the protocol to the ethics committee, while JM and TD have suggested important aspects for exercise protocol, functional measures and quality of life. The manuscript was prepared by DH, JM, and TD. All authors revised the manuscript critically for important intellectual content and approved the final version to be published.

## Pre-publication history

The pre-publication history for this paper can be accessed here:

http://www.biomedcentral.com/1471-2369/14/259/prepub
